# Accelerating Bone Healing With METTL3 Overexpressed Adipose‐Derived Stem Cells in Osteoporotic Rats

**DOI:** 10.1111/cpr.70029

**Published:** 2025-03-24

**Authors:** Hui Tang, Zhenzhen Chen, Lu Zeng, Yuping Xie, Daowen Luo, Shuanglin Peng, Fangzhi Lou, Tianli Wu, Jingang Xiao

**Affiliations:** ^1^ Department of Oral and Maxillofacial Surgery, The Affiliated Stomatological Hospital Southwest Medical University Luzhou China; ^2^ Department of Oral Implantology, The Affiliated Stomatological Hospital Southwest Medical University Luzhou China; ^3^ Luzhou Key Laboratory of Oral & Maxillofacial Reconstruction and Regeneration, The Affiliated Stomatological Hospital Southwest Medical University Luzhou China; ^4^ Department of Oral and Maxillofacial Surgery, The Affiliated Hospital Southwest Medical University Luzhou China

**Keywords:** METTL3, N6‐methyladenosine, OP‐ASCs, osteoporosis, Wnt signalling pathway

## Abstract

The treatment of postmenopausal osteoporosis (OP) presents a multifaceted challenge. Nonetheless, emerging research indicates a significant association between the N6‐methyladenosine (m6A) methylase METTL3 and osteogenesis in OP. To investigate Mettl3's impact on osteogenic potential and the underlying molecular mechanisms, an OP rat model was established via ovariectomy (OVX). Osteoporotic adipose‐derived stem cells (OP‐ASCs) were then isolated. Results indicated a significant downregulation of Mettl3 expression in OP‐ASCs. Subsequently, OP‐ASCs were transfected with overexpressed Mettl3 lentivirus and treated for Dickkopf‐related protein‐1 (DKK1). Overexpression of the Mettl3 gene led to increased levels of osteogenic factors. DKK1 attenuated osteoblastic differentiation capacity in the Mettl3 overexpression group by inhibiting the Wnt signalling pathway. Consistent results were observed in vivo experiments. In conclusion, overexpression of Mettl3 promotes osteogenesis in OP‐ASCs by activating the Wnt/β‐catenin pathway.

## Graphical abstract

METTL3 promotes osteogenic differentiation of osteoporotic adipose‐derived stem cells and accelerates the repair of cranial defect areas in osteoporotic rats by activating the Wnt signalling pathway.

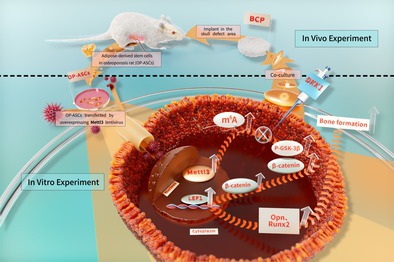

## 1 Introduction

Osteoporosis (OP) is a medical condition defined by the reduction in bone density and weakened bone structure, resulting in elevated fracture susceptibility and overall bone fragility [1, 2]. Primary OP encompasses postmenopausal OP and senile OP, while secondary OP is primarily caused by diseases affecting bone metabolism and other factors [3, 4]. Due to decreased oestrogen levels, postmenopausal women experience alterations in bone microarchitecture, increased bone fragility and reduced bone strength [5, 6]. The incidence of OP in postmenopausal women is also on the rise, significantly impacting their quality of life and ability to work. Addressing bone defects in postmenopausal OP has become a pressing clinical challenge.

In recent years, bone tissue engineering has attracted a great deal of attention in the OP therapeutic field [7, 8]. This approach utilises principles and technologies from engineering and cell biology to construct new bone tissue replacements in vitro using seed cells, aiming to repair and restore the morphology and function of damaged bone tissue. The ideal seed cells for bone tissue engineering are stem‐type cells capable of proliferating and differentiating into bone tissue [9, 10]. Currently, mesenchymal stem cells (MSCs) harvested from the bone marrow, known as BMSCs, and those sourced from adipose tissue, referred to as adipose‐derived stem cells (ASCs), are frequently employed as the primary cellular agents in a range of stem cell applications [11, 12]. While BMSCs are considered ideal seed cells, they are scarce and challenging to obtain from postmenopausal OP patients [13]. ASCs, obtained from autologous fat, present fewer ethical concerns [14]. The ease of acquiring ASCs, regardless of donor area, is a significant advantage over BMSCs, making ASCs promising for clinical bone tissue repair. However, recent research suggests that osteoporotic ASCs (OP‐ASCs) have a lower osteogenesis capacity than ASCs [15].

One crucial signalling mechanism controlling osteogenesis is the Wnt pathway, which regulates differentiation, migration, and other physiological activities during cell development [16, 17]. The Wnt pathway comprises WNT proteins, β‐CATENIN protein, other molecules, ligands and receptors, divided into canonical and noncanonical branches, with current research focusing on the former [18, 19]. Studies indicate that morusin activates the Wnt/β‐catenin pathway, enhancing the osteogenic potential in BMSCs from wistar rats and improving therapeutic efficacy in OP treatment [20]. Additionally, research suggests that Runx1 preserves adult bone homeostasis by enhancing Wnt/β‐catenin signalling pathways [21].

N6‐methyladenosine (m6A) is considered to be a major form of mRNA revision in animal cells, characterised by the presence of a methyl group at the N6 position of adenosine residues within the mRNA molecule [22, 23]. Molecules involved in RNA methylation mainly fall into three categories: ‘writers’, ‘erasers’ and ‘readers’. Writers, such as METTL3 and METTL14, mediate RNA methylation, while erasers like FTO and ALKBH5 remove RNA methylation. Readers recognise and bind to RNA methylation, such as proteins with the YTHDF domain [24, 25]. The m6A methyltransferase METTL3 and demethyltransferase FTO are crucial for adipogenic and osteogenic differentiation processes, playing significant roles in OP pathology [26]. Studies show that conditional Mettl3 knockdown in mouse BMSCs results in decreased bone mass and elevated fat in the bone marrow [27]. Another study indicates that Mettl3 knockout leads to decreased expression of osteogenic factors, negatively affecting osteogenic differentiation and bone mineralization [28]. These findings suggest that Mettl3 expression positively correlates with osteogenesis and negatively with adipogenesis.

While it is reasonable to propose that the decreased osteogenic ability of OP‐ASCs is linked to reduced Mettl3 expression, the molecular mechanisms involved require further exploration. With the present study, we are investigating the essential effects of Mettl3 on the prospective regulation of the Wnt signalling cascade and the process of bone formation in OP‐ASCs.

## 2 Materials and Experimental Procedure

### 2.1 OP Model Establishment in SD Rats

**FIGURE 1:**
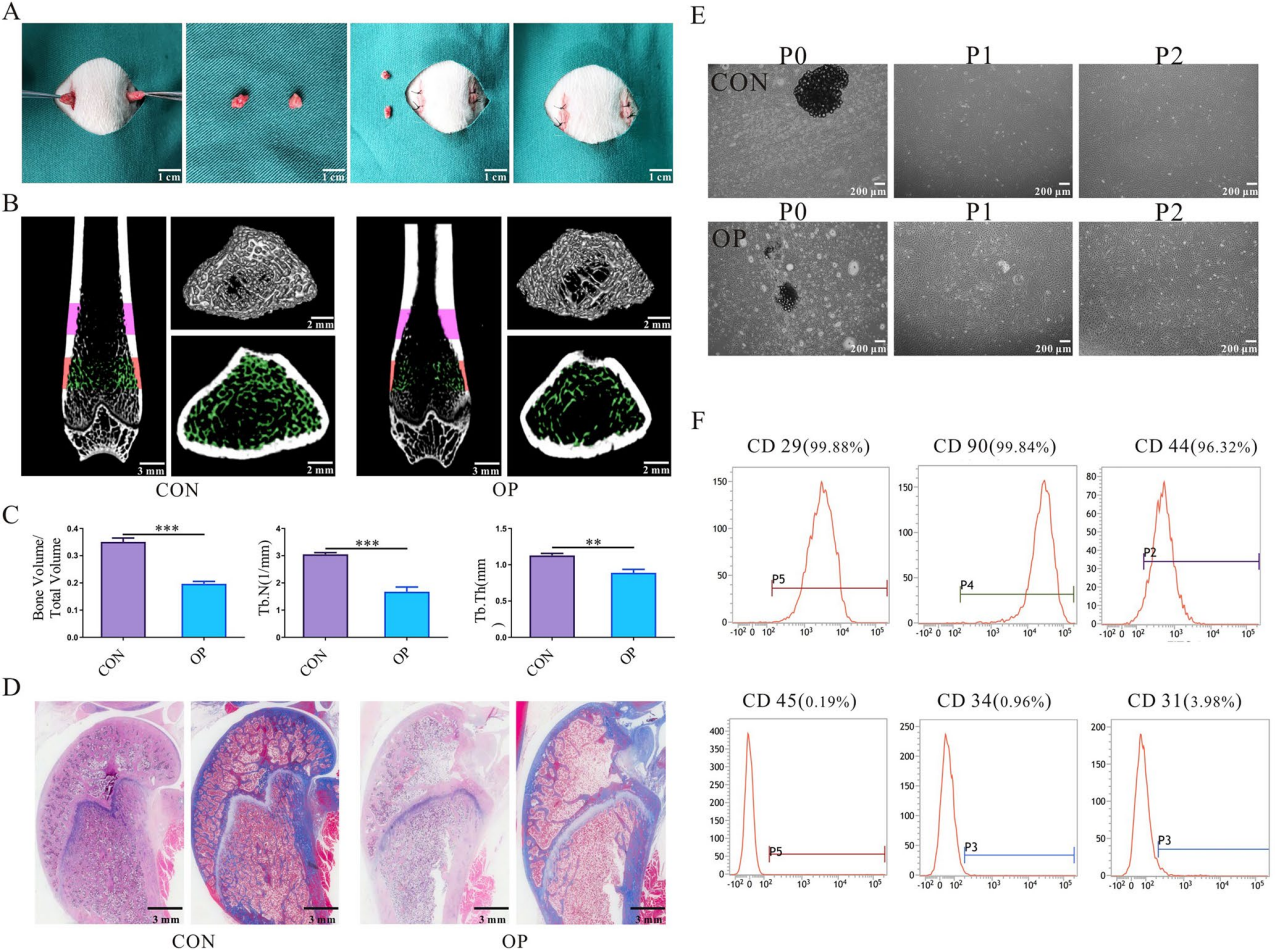
Establishment of an osteoporosis rat model and culture of ASCs. (A) A model of OP was created in female SD rats through the surgical removal of both ovaries. (B, C) Micro‐CT identification of osteoporosis modelling. (D) The rat femur's H&E and Masson staining identification of osteoporosis modelling. (E, F) Culture and characterisation of ASCs. (**p < 0.01, ***p < 0.001).

The 4‐week‐old female SD rats were acquired from the Animal Experiment Center of Southwest Medical University. All the surgeries were approved by the ethics committee of Southwest Medical University. Rats were randomised into experimental (OP group) and control groups (CON group). The rats were put in a device for tiny animals to be sedated, and isoflurane was used to make them unconscious. After the rats were anaesthetised, body weight was weighed, and 0.05 mL/g of 1% pentobarbital was administered intravenously to the animals' abdominal cavity. Their body hair (back and both abdomens) in the operation area was shaved, and the rats were disinfected with 75% alcohol by lying prone on the operating table and spreading surgical towels. The incision was made 0.8–1 cm on either side of the spine at the maximum expansion of the abdomen on both sides. The incision was ~0.8 cm long and parallel to the spine. The skin, subcutaneous fascia, fat layer, and muscle layer were incised separately to expose organs and creamy fat. The ovary was pulled out gently with small forceps, and the fallopian tube was clamped with vascular forceps at 3 mm from the fixed end of the ovary. The upper end of the hemostat was ligated with a 0‐gauge silk ligature, leaving a 0.5 mm end of the fallopian tube. After the ovary was completely removed, the hemostatic forceps were loosened. Gently place the pulled tissue back into the abdominal cavity, checking for bleeding and loose threads, and suture the layers of tissue successively (Figure 1A). The CON group received the same procedure, but the same amount of adipose tissue was removed without damage to the ovaries or fallopian tubes. Rats were disinfected with iodophor and kept warm. Then, rats were fed continuously for 3 months.

### 2.2 Micro‐CT (Micro‐Computed Tomography) Analysis

After the samples were collected, they were immersed in 4% paraformaldehyde for fixation and analyzed by micro‐CT. The main observation area of the femur is from the distal end of the femur to the epiphysis, about 1 cm. The main observation area of the skull is the central material. Bone volume (BV), tissue volume (TV), bone surface area (BS), bone volume fraction (BV/TV), trabecular number (Tb.N), trabecular thickness (Tb.Th), and trabecular separation (Tb.Sp) were analysed. A statistical analysis was performed after the analysis was completed.

### 2.3 Haematoxylin and Eosin (H&E) and Masson's Staining

After micro‐CT scanning, 4% paraformaldehyde was used to fix the samples. After 48 h of fixation, the specimens were immersed in a decalcifying solution and then incubated at 37°C within a water bath for 3 days. Subsequently, they were thoroughly washed under a continuous flow of tap water for a duration of 48 h. Then, the samples were dehydrated, embedded, sliced (5 mm), and air‐dried overnight at 37°C. Finally, H&E and Masson stains were used to colour the samples.

### 2.4 Identification of ASCs

Under sterile conditions, a piece of adipose tissue about 1.0 cm in size was taken from the rats, and it was cut into a paste and evenly covered the bottom of the 25‐T cell culture flask. The cell flask was placed upside down for 8 min. A total of 3 mL of complete culture medium was introduced into it. To obtain primary culture cells, the tissue fragments were cultivated in incubators. Cell fusions were between 80% and 90%; the cells began to pass through, and the first generation of cells was obtained. According to this method, third‐generation cells were used for the experiment.

The P3‐generation ASCs in good condition were digested, centrifuged and resuspended in PBS. One group of test‐tube cells was stained with fluorophore‐labelled antibodies against CD 29, CD 44, CD 45, CD 90, CD 31 or CD 34, and the other group of test‐tube cells without fluorophore antibodies was used as the control. A FACSCalibur flow cytometer examined the cells.

### 2.5 Quantitative Polymerase Chain Reaction (qPCR)

RNA Extraction Kit (Tiagen, China) was used to obtain total RNA from samples. The mRNA was then extracted with the Dynabeads mRNA Purification Kit (Thermo, Waltham, USA); nucleic acid transfer was performed on Hybond‐N+ (SIGMA, Maryland, USA) membranes, and the mRNA was crosslinked to Hybond‐*N*+ membranes using a UV crosslinker (Stratalinker). The m6A antibody (Synaptic Systems, Shanghai, China) was placed on the membranes overnight after being blocked in 5% skim milk for 1 h. The membranes were treated with goat anti‐rabbit IgG‐HRP (Signalway Antibody, Maryland, USA) for 1 h. Images were obtained after rinsing using an enhanced ECL detection system.

In addition, using Thermo's RevertAid First Strand cDNA Synthesis Kit, RNA was reverse transcribed to create cDNA. To detect cDNA, qPCR was employed in a PCR apparatus (Bio‐RAD USA). Table 1 lists the genes that were detected, which also included osteopontin (*Opn*) and *Runx2*, *β‐Catenin*, lymphoid enhancer factor 1 (*Lef1*), glycogen synthase kinase‐3β (*Gsk‐3β*), and methyltransferase‐like 3 (*Mettl3*). An internal reference was glyceraldehyde phosphate dehydrogenase (*Gapdh*).

**TABLE 1:** Primer sequences for related genes.

Gene	GenBank no.	Primers (5′‐3′)
*Gapdh*	NM_017008.4	*Gapdh f* TTTGAGGGTGCAGCGAACTT *Gapdh r* ACAGCAACAGGGTGGTGGAC
*Opn*	NM_012881.2	*Opn f* CCAGCCAAGGACCAACTACA *Opn r* CTGCCAAACTCAGCCACTTTC
*Runx2*	NM_001278483.1	*Runx2 f* GAACCAAGAAGGCACAGAC *Runx2 r* AATGCGCCCTAAATCACTG
*Mettl3*	NM_001024794	*Mettl3 f* CTCAGATCTCGCCTTAACCTTGCC *Mettl3r* GACAGCTTGGAGTGGTCAGCATAG
*β‐catenin*	NM_053357.2	*β‐catenin f* ACTCTAGTGCAGCTTCTGGGTTCTG *β‐catenin r* CTCGGTAATGTCCTCCCTGTCA
*Lef1*	NM_130429.1	*Lef1 f* CAGACCTGTCACCCTTCAGC *Lef1 r* GTGAGACGGATTGCCAAACG

### 2.6 Western Blot Assay (WB)

Samples were collected on the 3rd and 5th days of the osteogenic induction process; the overall protein content was isolated utilising the Keygen Biotech Total Protein Extraction Kit. After being separated on 12% SDS‐PAGE, proteins were moved to membranes made of polyvinylidene fluoride (Bio‐Rad, USA). Then, the PVDF membranes and related antibodies were incubated overnight at 4°C, including GAPDH (ab181602), RUNX2 (ab92336), OPN (ab91655), β‐CATENIN (ab32572), GSK3β (ab32391), METTL3 (ab195352) (Abcam, UK) and LEF1 (2230p), P‐GSK‐3β (5558) (Cell Signalling Technology, USA). After 24 h, the membranes were rinsed thoroughly with TBST three times for 10 min; then diluted secondary antibodies (Beyotime, China) were added and incubated for 1 h. The membranes were rinsed. The membrane surface liquid was aspirated and immersed in ECL luminescent solution (Affinity, Jiangsu, China) for 20 s. Then, the results of the membrane were observed.

### 2.7 Immunofluorescence of Osteogenesis‐Related Proteins

After culturing in osteogenic induction medium (OriCell, RAXMD‐90021, China), cells were fixed in 4% paraformaldehyde. 0.5% Triton‐100 (Triton‐100:PBS = 200:1) was washed three times for 10 min each time. Freshly prepared goat serum (PBS:goat serum = 20:1) was added at 37°C and incubated for 1.5 h. After the incubation, the corresponding primary antibodies for OPN and RUNX2 (PBS:primary antibody = 100:1) were included and kept at 4°C for the whole night. The sample was warmed on a shaker for 30 min on the second day. After rewarming, a fresh secondary antibody (PBS:secondary antibody = 200:1) was added and incubated for 1 h. Then the currently prepared phalloidin (FITC:PBS = 1:100) at 37°C for 30 min. Then, DAPI (DAPI:PBS = 1:1000) was coated for 15 min and rinsed. Finally, 10% glycerol was added to mount the slides, and a small quantity of an anti‐quenching agent was added to the glycerol. Images were obtained under a confocal microscope as soon as possible.

### 2.8 Staining With Alkaline Phosphatase (ALP) and Alizarin Red (ARS)

Following an osteogenic induction for 3 and 5 days, cells were fixed with 4% paraformaldehyde for 0.5 h. 1 mL of the BCIP/NBT staining working solution (Sangon Biotech, China) was mixed and incubated in the dark for 1 h. After 1 h of incubation, it was washed twice with distilled water to stop the colour‐developing response. The staining outcomes were seen and noted.

Following an osteogenic induction for 21 days, the samples were preserved for 30 min in 4% paraformaldehyde. 0.5 mL of ARS (Cyagen, China) was kept in each well for 5 min.

### 2.9 Creation of OP‐ASCs‐Seeded Scaffolds for BCP

A biphasic calcium phosphate scaffold (BCP) was provided by the Biomaterials Engineering Research Center of Sichuan University. According to the grouping, each BCP scaffold was inoculated with about 1 × 10^5^ cells, respectively, and cultivated for 48 h in an osteogenic induction medium to lay the foundation for the subsequent in vivo experiment.

### 2.10 Establishment of a Rat Skull Critical‐Size Bone Defect Model and In Vivo Implantation of OP‐ASCs‐Seeded BCP Scaffolds

The OP group's rats were allocated into three groups at random. Under sterile conditions, each rat's skull formed an 8‐mm‐diameter circular defect. The cranial surgery area was routinely prepared and sterilised. A sterile, perforated towel was applied. The midline of the skull was first determined, and then a skin incision about 2 cm long was made along the longitudinal direction. Then the skin was bluntly separated and incised in a ‘T’ shape, and the periosteum and the skull were exposed. It was first cut with an 8‐mm‐diameter electrosurgical amputator and then removed slowly. Throughout the whole grinding process, it was necessary to moisten and cool the material with sterile normal saline. During the operation, it must be well protected. The meninges and the large blood vessels beneath them, as well as the periosteum on the inner and outer surfaces of the skull, were completely removed to form an 8‐mm cranial defect. After fixation of the periosteum and BCP, the skin and muscular layers were sewn separately, and the whole procedure should be performed aseptically to prevent infection. After the surgery, the rats were allowed to drink, eat and feed freely under suitable conditions.

### 2.11 Statistical Analysis

Each experimental group's findings were replicated on a minimum of three separate occasions. The data analysis was carried out using SPSS 20.0. The data analysis method used was the student's *t*‐test or one‐way ANOVA. Significance was assigned to the difference if *p* < 0.05.

## 3 Results

### 3.1 Establishment of an OP Model and Culture and Identification of OP‐ASCs

OP models were established in SD female rats by bilateral OVX (Figure 1A). The model was established after 3 months, and the femurs of the two groups of rats were scanned by micro‐CT. The femoral marrow cavity of the OP group was significantly larger. Cortical bone thinning, bone trabeculae were sparse and the number of trabeculae was significantly reduced (Figure 1B). The femoral (BV/TV, Tb.N and Tb.Th) was substantially lower in the OP group (Figure 1C). The rat femur's H&E and Masson staining findings revealed that there were considerably fewer trabecular bones in the OP group, the arrangement was disordered, a larger bone marrow cavity was present, and the cortical bone became thinner (Figure 1D).

A small number of spindle‐shaped cells could be seen crawling out of the tissue block on the sixth day. After about 3 days, the cells covered the bottom of the flask, and the cells were spindle‐shaped and gradually arranged in a whirlpool shape. During the whole process of primary culture, passage and cell recovery, in comparison to the CON group, the OP group's cell condition and growth rate were poorer. The two groups of cell cultures are shown in Figure 1E. Findings of the P3 generation ASCs’ isolation and culture using flow cytometry showed that the cellular surface markers CD 29 and CD 90 were expressed at high levels, and CD 45 and CD 34 were expressed at low levels (Figure 1F). The results showed that ASCs are stem cells, excluding the possibility of blood‐derived cells. Thus, it can be shown that high‐purity ASCs can be obtained by extracting rat inguinal fat using the tissue block apposition method.

### 3.2 Downregulated Osteogenesis of OP‐ASCs

**FIGURE 2:**
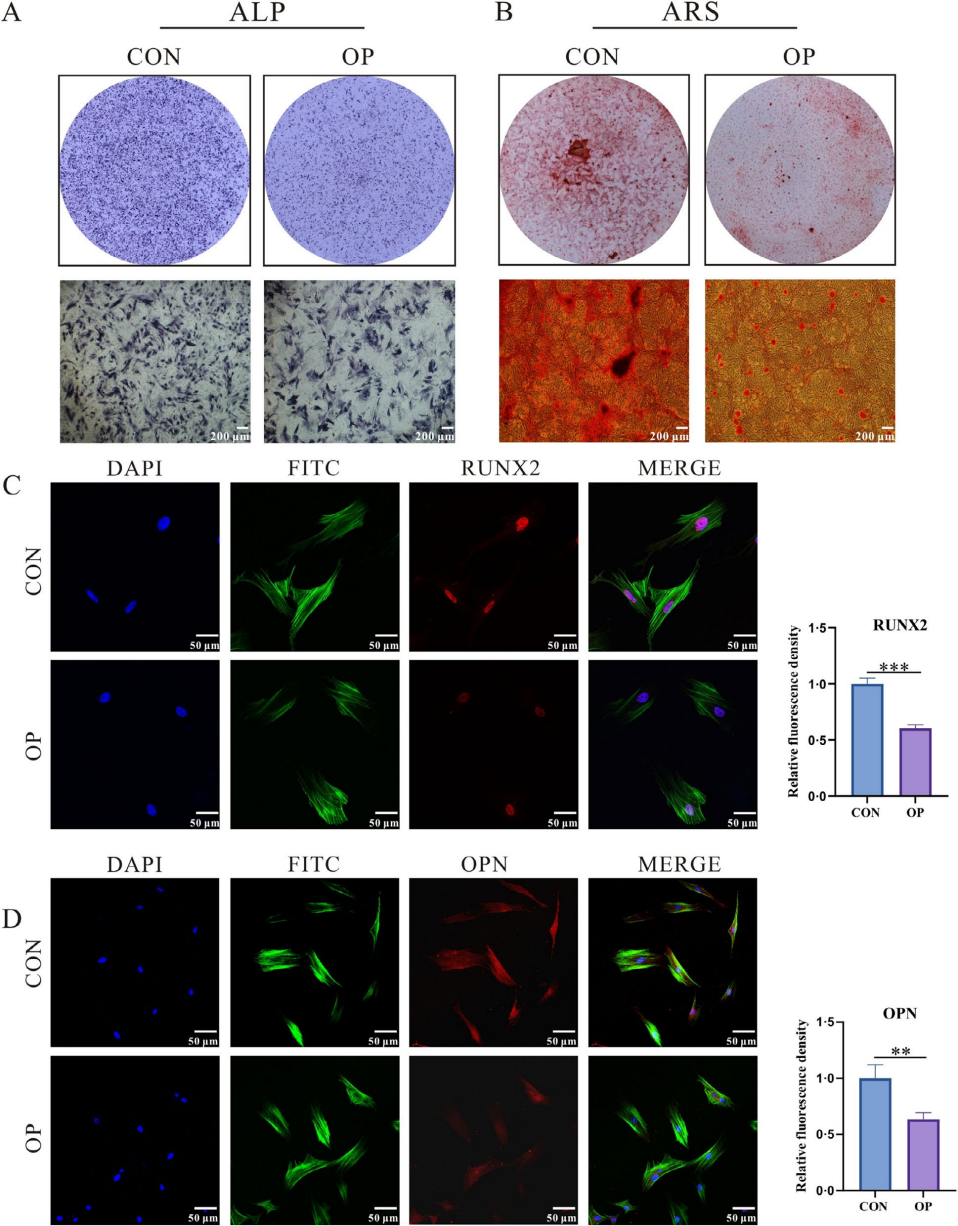
Detection of Differences in Osteogenic Capacity between ASCs and OP‐ASCS. (A) ALP staining was conducted after 5 days of osteogenic induction. (B) ARS staining was performed at 21 days of osteogenic induction. (C, D) The fluorescence of OPN and RUNX2 proteins (**p < 0.01, ***p < 0.001).

**FIGURE 3:**
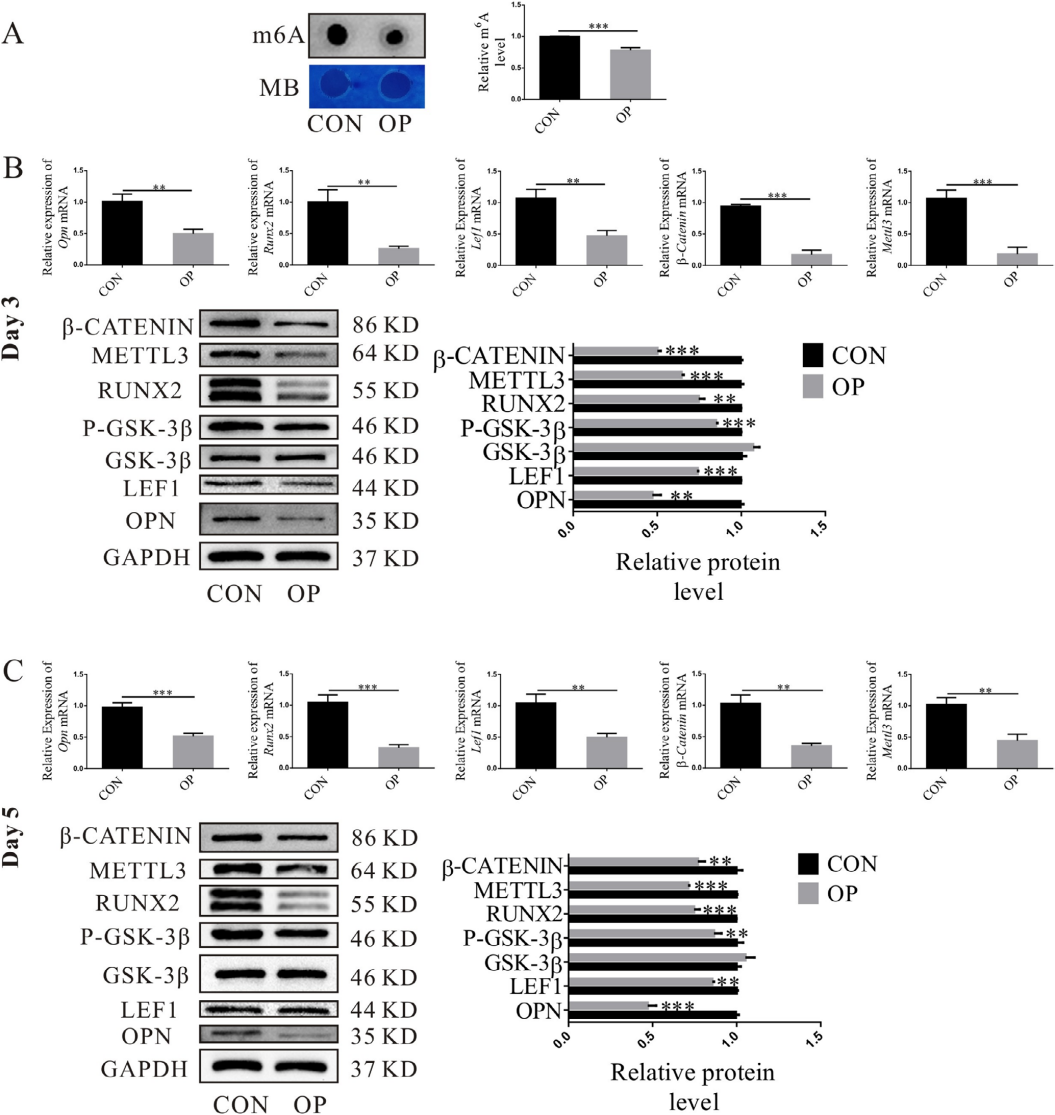
RNA methylation alterations may be connected to Wnt signalling pathway inhibition. (A) mRNA expression level of the m6A methylation. (B, C) The typical osteogenic and Wnt‐related factors expression was monitored at both 3 days and 5 days (**p < 0.01, ***p < 0.001).

The two groups of cells were subjected to q‐PCR and WB detection following an osteogenic induction of three and 5 days, respectively. The outcomes demonstrated a higher level of Opn and Runx2 expression in the CON group (Figure 3B,C). The difference was statistically significant. ALP staining was carried out after osteogenic induction for 5 days, and the outcomes revealed that the OP group stained significantly lighter than the CON group (Figure 2A). Following 21 days of treatment with an osteoinductive solution, ARS was applied to cells. The mineralized nodules within the OP group appeared fewer and exhibited a paler staining intensity in comparison to the CON group (Figure 2B). The osteogenesis‐related proteins were detected by immunofluorescence after 3 days in the osteogenic induction solution of the two groups. The findings demonstrated that OPN and RUNX2 fluorescence intensity was noticeably higher in the CON group (Figure 2C,D).

The two groups of cells were subjected to q‐PCR detection following the osteogenic induction for 3 and 5 days. *Lef1* and *β‐catenin* genes, which are connected to the Wnt signalling system, expressed more in the CON group, and a significant statistical variance was observed (Figure 3B,C). Meanwhile, the WB results also showed that after 3 days of osteogenic induction (Figure 3B) and 5 days (Figure 3C), β‐CATENIN and P‐GSK‐3β proteins were higher than those of the OP group. While GSK‐3β protein expression was comparable to that of the OP group, the variation lacked statistical significance.

### 3.3 Inhibition of the Wnt Signalling Pathway in OP‐ASCs May Be Related to Changes in RNA Methylation Levels

**FIGURE 4:**
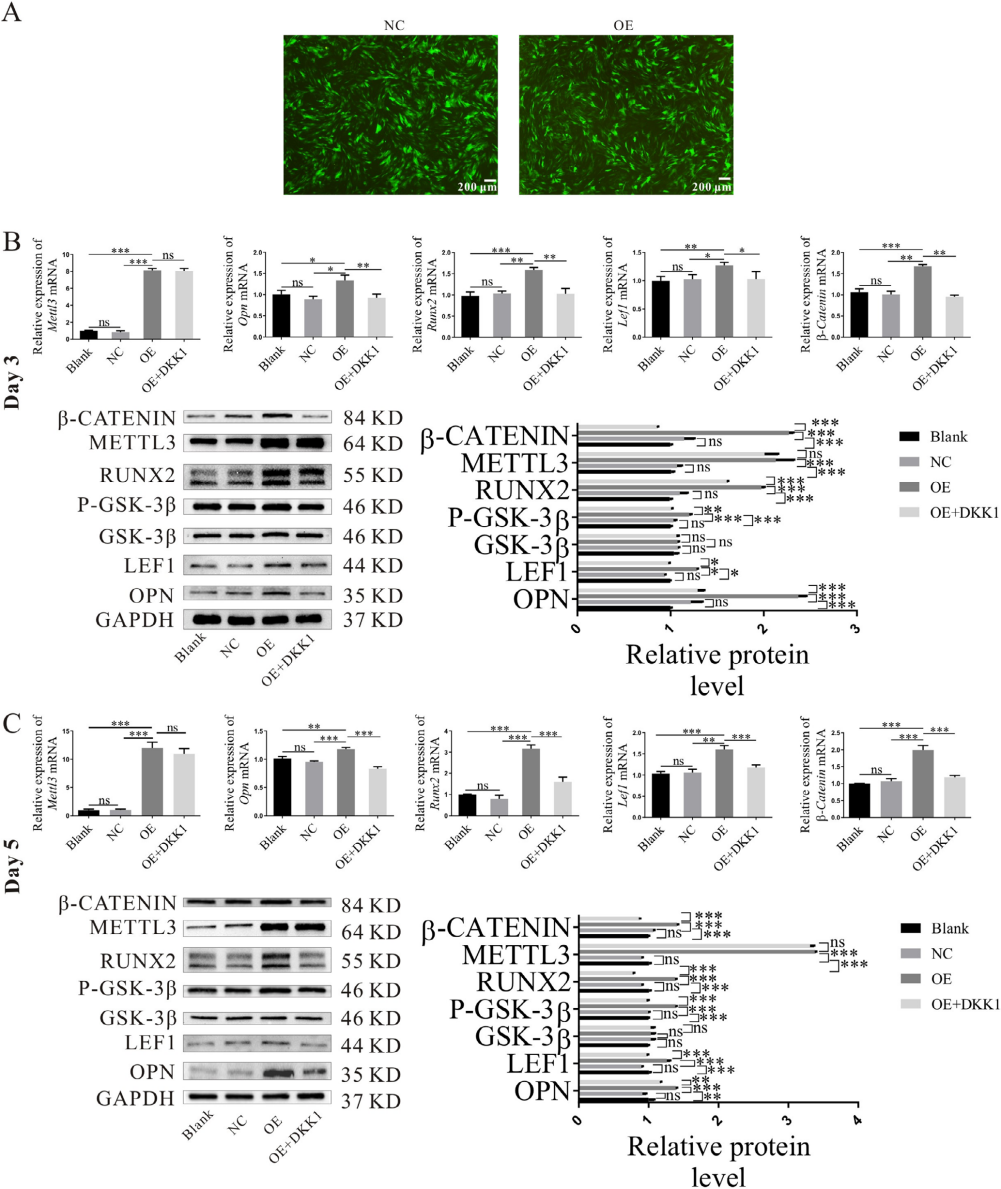
*Mettl3* gene overexpression activates the Wnt pathway and promotes osteogenesis. (A) Transfection effect of two groups of cells with an MOI value of 80. (B, C) The gene and protein expression levels of Mettl3, Opn, Runx2, β‐catenin, p‐GSK‐3β and Lef1 following 3 days and 5 days of osteogenic induction (*p < 0.05, **p < 0.01, ***p < 0.001).

The two groups of cells were detected by q‐PCR and WB following an osteogenic induction of 3 and 5 days, respectively. The m6A methylation expression level of mRNA was lower in OP‐ASCs (Figure 3A). In comparison to the OP group, the CON group had increased expression levels of the m6A RNA methylase Mettl3 gene and protein (Figure 3B,C). Different MOI value gradients were set for the two groups of lentiviruses. The findings revealed that the fluorescence expression was the strongest, as the MOI value was 80. The 80 MOI value was used for subsequent virus transfection (Figure 4A).

**FIGURE 5:**
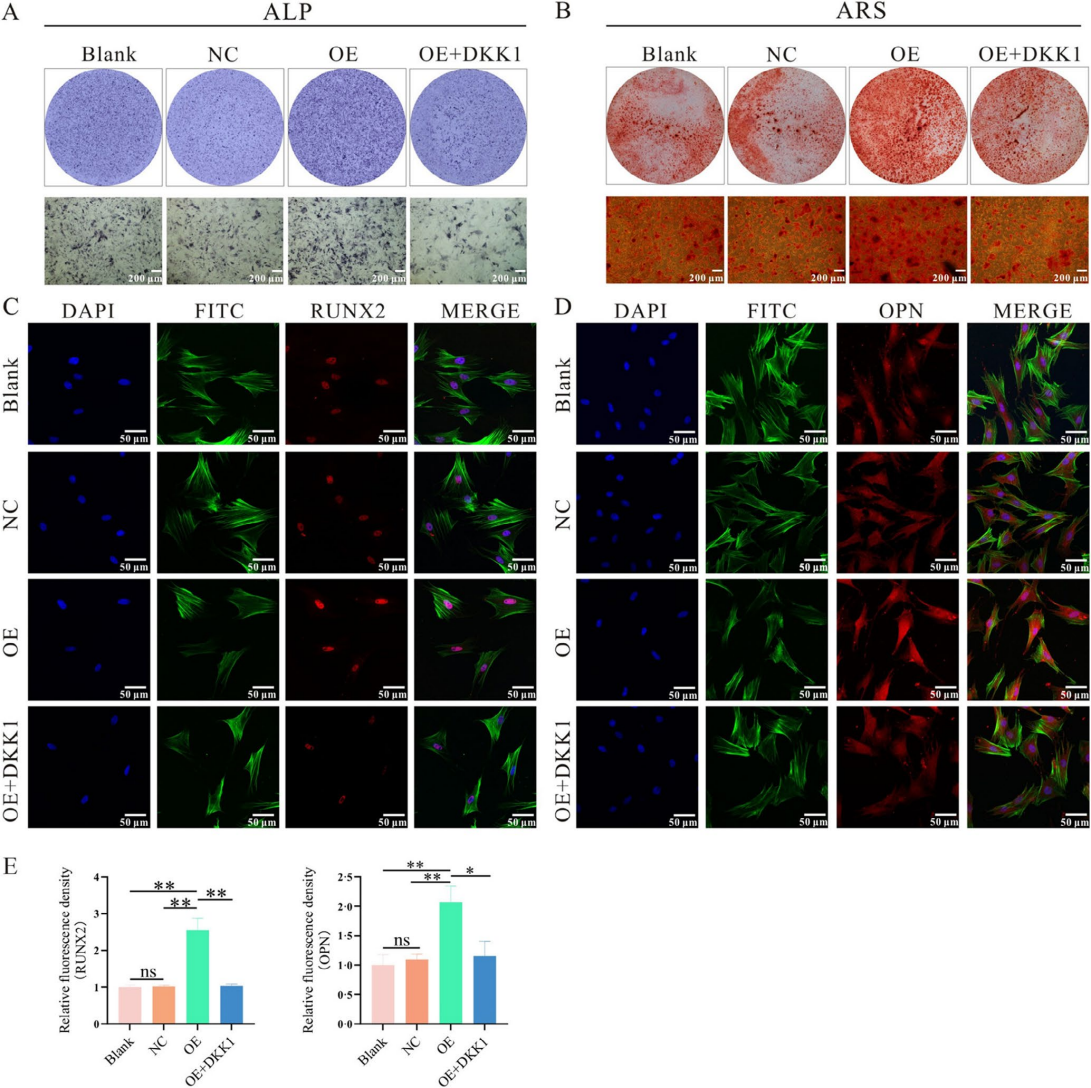
*Mettl3* gene overexpression promotes osteogenesis in vitro. (A) ALP staining was performed after overexpression of Mettl3. (B) The mineralized nodules (C, D) The fluorescence expression of OPN and RUNX2 proteins. (E) Semi‐quantitative analysis of fluorescence intensity by ImageJ (*p < 0.05, **p < 0.01, ***p < 0.001).

The findings revealed that the gene and protein expressions of the OE group (*Mettl3* overexpression) and the OE + DKK1 group (*Mettl3* overexpression+DKK1) were greater than those of the Blank group (no lentivirus) and NC group (lentivirus without *Mettl3*). Statistical significance was found in the difference. The expression levels of the blank control group and the empty virus control group were similar, and the variation lacked statistical significance (Figure 4B,C). The q‐PCR results showed that 3 days and 5 days after osteogenic induction, the expression of *β‐catenin*, *Lef1*, *Opn*, and *Runx2* genes in the OE group was far higher than in other groups, and the expression of the OE + DKK1 group was much lower than in the OE group. The difference revealed statistical significance (Figure 4B,C). The WB results also revealed that the protein expressions of β‐CATENIN, P‐GSK‐3β, OPN, and RUNX2 in the OE group were far higher than in other groups, and the OE + DKK1 group was much lower than in the OE group (Figure 4B,C). The expression of the GSK‐3β protein was comparable among the four groups, and the difference lacked statistical significance. The cells in the four groups underwent osteogenic induction for 5 days. The outcomes of the ALP staining revealed that, compared to the first two groups, the OE group's staining was noticeably darker, while OE + DKK1 had lighter staining than the *Mettl3* overexpression group (Figure 5A). ARS indicated that the calcium nodules in the OE group were significantly increased, and the colouring was deeper than in other groups (Figure 5B). The findings also demonstrated that OPN and RUNX2 protein fluorescence intensity in the OE group was noticeably higher than in other groups (Figure 5C–E).

### 3.4 In Vivo Validation of Mettl3 to Regulate the Osteogenic Ability of OP‐ASCs in Rats

**FIGURE 6:**
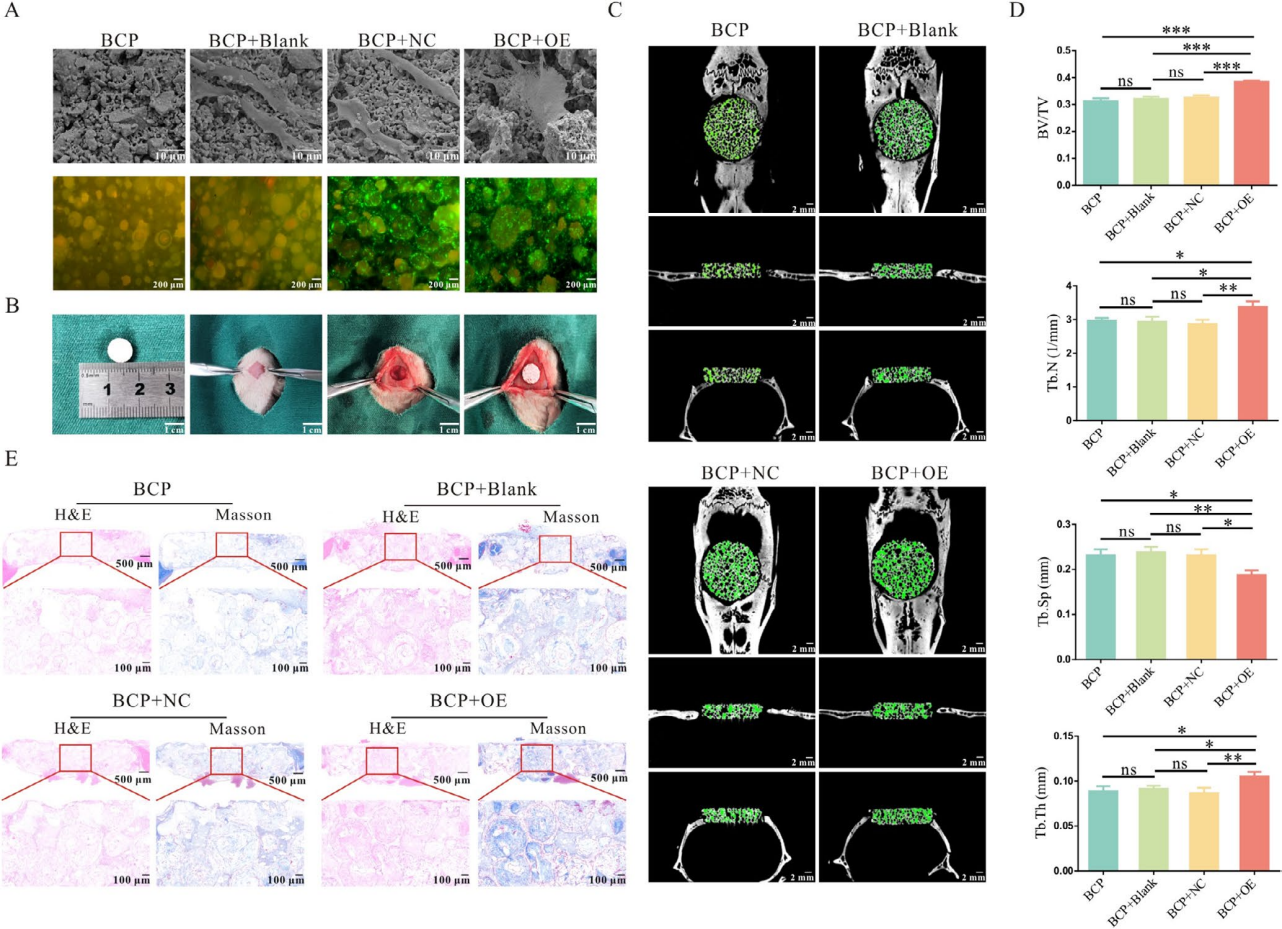
*Mettl3* gene overexpression promotes osteogenesis in vivo. (A) Scanning electron microscopes and fluorescence microscopes. (B) In OP rats, ASC‐seeded BCP scaffolds were implanted in a critical‐sized calvarial bone defect. (C, D) The Micro‐CT scan results of the SD rat. (E) The Masson and H&E staining results of rat calvaria (*p < 0.05, **p < 0.01, ***p < 0.001).

We made OP‐ASCs‐seeded BCP scaffolds to further assess Mettl3's osteogenic function in vivo. Scanning electron microscopes and fluorescence microscopes revealed that cells in the BCP + B group, the BCP + NC group, and the BCP + OE group adhered to the BCP scaffolds (Figure 6A). Then, we introduced scaffolds, pre‐colonised with ASCs, into a critical‐sized calvarial bone defect model in OP rats (Figure 6B). Micro‐CT and histological staining detected the repair of the rat skull defects for 8 weeks postoperatively. The micro‐CT scan revealed that BV/TV, Tb.N, and Tb.Th increased in the BCP + OE group compared with other groups. However, Tb.Sp was decreased, and the differences were all statistically significant (Figure 6C,D). Results of histological staining revealed that the BCP + OE group had notably higher degrees of fibrosis and mineralization of new bone compared to other groups. This was in line with the findings of the analysis of the micro‐CT scan data (Figure 6E). These findings concluded that Mettl3 regulated OP‐ASCs' osteogenesis in vivo. The reduced ability of OP‐ASCs to differentiate into osteoblasts was related to the lower expression of Mettl3.

## 4 Discussion

It has been reported that more than one‐quarter of postmenopausal women would have varying degrees of OP symptoms [29, 30]. These symptoms are manifested as increased bone fragility and eventually lead to fractures, which have significant consequences for patients. They cause pain and burden to society. In order to conduct research on postmenopausal OP in depth, it is crucial to establish relevant models of the disease. The researchers established an OP model by removing the bilateral ovaries of SD female rats. The method has been regarded as a classical model for establishing postmenopausal OP with a high modelling rate and good repeatability [31, 32]. In this experiment, female SD rats were also selected to establish an OP model by removing bilateral ovaries, and the success of model establishment was identified 3 months after the operation. Micro‐CT scans can clearly show the three‐dimensional anatomical structure of rat bones, and the changes in bone trabeculae can also be observed [33]. Our micro‐CT scan results showed that BV/TV, Tb.N, and Tb.Th in the OP group were inferior to the CON group's measurements. Tb.Sp increased in the OP group. In addition, histological staining results revealed that there were considerably fewer bone trabeculae in the OP group. The bone structure exhibited irregularities and disparities, with a noticeable reduction in the thickness of the cortical bone and an enlargement of the interstitial spaces within the skeletal framework. These were in line with the results of the micro‐CT scan analysis and proved that the OP model for the OP group was successfully established.

Similar to BMSCs, ASCs are essential for tissue engineering since they can differentiate into adipose, bone, cartilage, and other tissues [34]. Compared with BMSCs, a major advantage of ASCs is that they are easily accessible and can be obtained repeatedly. Large amounts of fat tissue can be harvested through a less invasive procedure with less impact on the harvested area [35]. ASCs have immunosuppressive properties and low immunogenicity, which also make them promising for applications in tissue regeneration. In addition, it has been shown that ASCs are later aging than BMSCs, possess a stronger cell division capacity, and are ideal cellular therapies for chronic and persistent diseases [36, 37]. It has been shown that OVX rats' osteogenic capacity is much inferior to that of normal rats [38, 39]. In contrast to the CON group, we discovered that the Runx2 and Opn were decreased in the OP rats. ALP activity and the quantity of calcium nodules decreased in the OP group. Our experimental findings are consistent with those reported in the literature. However, it is yet unknown how OP‐ASCs' lower osteogenic ability is caused, and further study is required.

Through controlling osteoblast development and function, the Wnt/β‐catenin signalling pathway is essential for maintaining bone mass [40]. According to studies, chrysosplenetin stimulates hBMSCs' ability to produce osteoblasts by turning on the Wnt pathway [41]. Additionally, research has shown that the canonical Wnt pathway is necessary for MSC development into osteoblast‐lineage cells. Activating the Wnt signalling system in OP mice facilitates ASCs' effective bone‐forming abilities [42, 43]. Comparing OP‐ASCs to ASCs, it can be shown that the Wnt/β‐catenin signalling pathway is suppressed. Because of this, we surmise that this pathway's suppression is responsible for the lower osteogenic capacity of OP‐ASCs, while the precise mechanism is yet unknown.

M6A methylation is a ubiquitous epigenetic mark that is critical in the maintenance of cellular homeostasis. It is intricately related to the regulation of gene expression beyond transcription, cellular proliferation and developmental processes [44, 45]. Additionally, it is crucial for the metabolism and formation of bones. According to studies, the m6A methyltransferase METTL3 is crucial for MSC differentiation and adipogenesis. In swine BMSCs, the expression of Mettl3 is inversely linked with adipogenesis [46]. M6A alterations have a significant impact on OP in addition to being involved in bone growth. Studies have revealed that the pathological characteristics of OP in mice were brought on by the conditional deletion of the m6A methyltransferase Mettl3 in BMSCs. Increased bone marrow fat, decreased osteogenic differentiation capacity, and poorer bone production are all consequences of Mettl3 function loss [47]. In the complex pathway between adipogenic development and osteogenic differentiation, the m6A methyltransferase METTL3 is implicated and is of great significance to the pathological development of OP. We found the downregulation of Mettl3 in the OP‐ASCs group. Thus, we hypothesised that the diminished levels of METTL3 might be associated with the dampening of the Wnt signalling cascade. Employing a lentiviral approach to enhance METTL3 expression, we observed shifts in molecular markers pertinent to osteogenic processes and the Wnt/β‐catenin signalling axis. Notably, the group with elevated METTL3 levels demonstrated a significant upregulation of β‐catenin, Lef1 and P‐GSK‐3β, suggesting that METTL3 overexpression may stimulate the Wnt/β‐catenin pathway. Furthermore, the Mettl3‐overexpressing group exhibited higher levels of Opn and Runx2, which could imply a facilitative role of METTL3 overexpression in osteogenic differentiation. To substantiate the activation of the Wnt/β‐catenin signalling by METTL3 overexpression, DKK1 was introduced. The group treated with both Mettl3 overexpression and DKK1 exhibited a reduction in β‐catenin and Lef1 expression levels when juxtaposed with the Mettl3 overexpression group alone. Therefore, we can conclude that *Mettl3* gene overexpression activates this signalling pathway and promotes osteogenesis.

To find out whether *Mettl3* overexpression can promote osteogenesis in SD rats, we constructed a rat model with extreme calvarial defects. Studies have shown that rat calvarial defects of 8 mm in diameter could not heal by themselves [48]. We implanted BCP scaffolds containing OP‐ASCs into the calvaria of OP rats. Eight weeks following surgery, micro‐CT and histochemistry showed that the osteogenic capacity was enhanced by *Mettl3* gene overexpression in OP rats, and the overexpression of the *Mettl3* gene rescued the decline of osteogenic capacity in OP rats. Hence, we deduced that the elevated levels of *Mettl3* could orchestrate the osteogenic potential of ASCs within living organisms, aligning with the in vitro findings.

In summary, our findings demonstrate that METTL3 can regulate the differentiation potential of ASCs for osteogenesis in vitro and in vivo by modulating the Wnt signalling pathway. Overexpression of Mettl3 effectively prevents bone loss in osteoporotic rats. Our research on the mechanisms of OP rats may provide valuable insights for developing treatments for osteoporotic disorders. Therefore, METTL3 is expected to be a potential target for bone tissue regeneration and treatment of bone defects in OP and provides the theoretical basis for novel therapeutic strategies.

## Author Contributions

All authors made significant contributions to this study. Hui Tang conducted in vivo experiments and wrote the main manuscript. Zhenzhen Chen, Lu Zeng, and Yuping Xie performed in vitro experiments and analyzed the data. Daowen Luo, Shuanglin Peng, and Fangzhi Lou collected the data. Tianli Wu and Jingang Xiao conceived and designed the experiment, revised the manuscript, and provided funding. All authors have read and approved the final manuscript.

## Conflicts of Interest

The authors declare no conflicts of interest.

## Data Availability

The data that support the findings of this study are available from the corresponding author upon reasonable request.
